# Supplementary material for: Application of a Synthetic Microbial Community to Enhance Pepper Resistance Against Phytophthora capsici
Source: Plants (Basel). 2025 May 26;14(11):1625. doi: 10.3390/plants14111625 (PMC12157186; doi:10.3390/plants14111625)
Supplement: Supplementary file 1 [file plants-14-01625-s001.zip › plants-3596075-supplementary.pdf]

Figure S1.

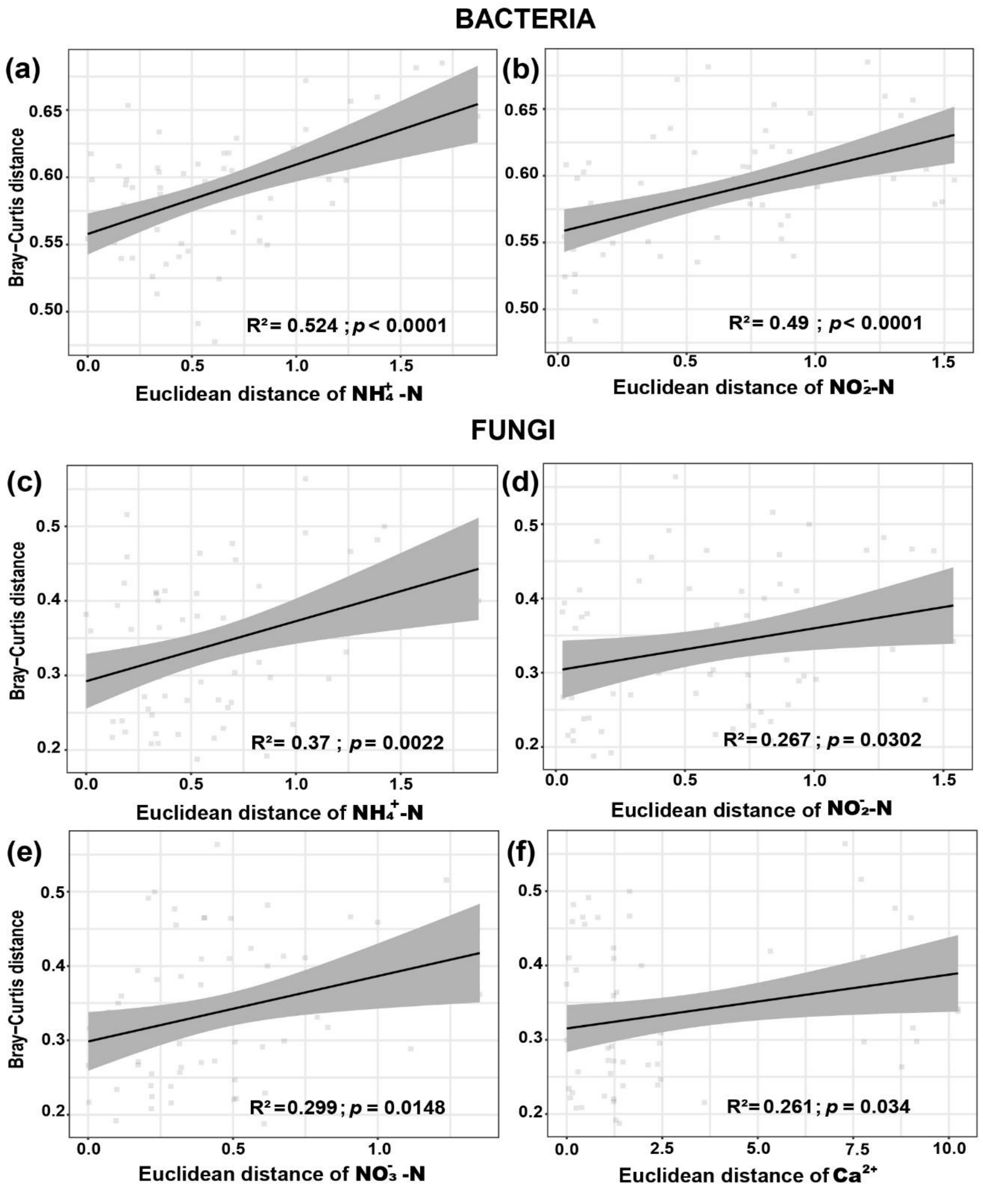

Figure S1. Correlation between Bray–Curtis dissimilarity of microbial communities and Euclidean distance of environmental parameters in the rhizosphere of pepper plants. (a, b) Correlation of bacterial community composition with NH_4^+-N and NO_2^--N, showing

significant positive relationships (R^2 = 0.524, p < 0.0001 and R^2 = 0.49, p < 0.0001, respectively). (c–f) Correlation of fungal community composition with NH_4^+-N, NO_2^--N, NO_3^--N, and Ca^{2+} (R^2 = 0.37, p = 0.0022; R^2 = 0.267, p = 0.0302; R^2 = 0.299, p = 0.0148; and R^2 = 0.261, p= 0.034, respectively). Shaded areas represent the 95% confidence intervals of the regression lines.

Table S1. Analysis of 16S rRNA gene sequences and comparison with accession iden tities retrieved from the NCBI database under 16S ribosomal RNA sequence category.

Strain	Accession number	Blast identification	% similarity	Blast accession number
T3	PP763279	*Bacillus sp.*	99.34	KC169801
T4	PP763424	*Flavobacterium anhuiense*	99.56	CP023642
T8	OR857502	*Cytobacillus firmus*	99.18	PP478111
T14	PP763444	*Streptomyces roseicoloratus*	99.93	MG266298
A6	PP763475	*Pseudomonas frederiksbergensis*	100	MK774798

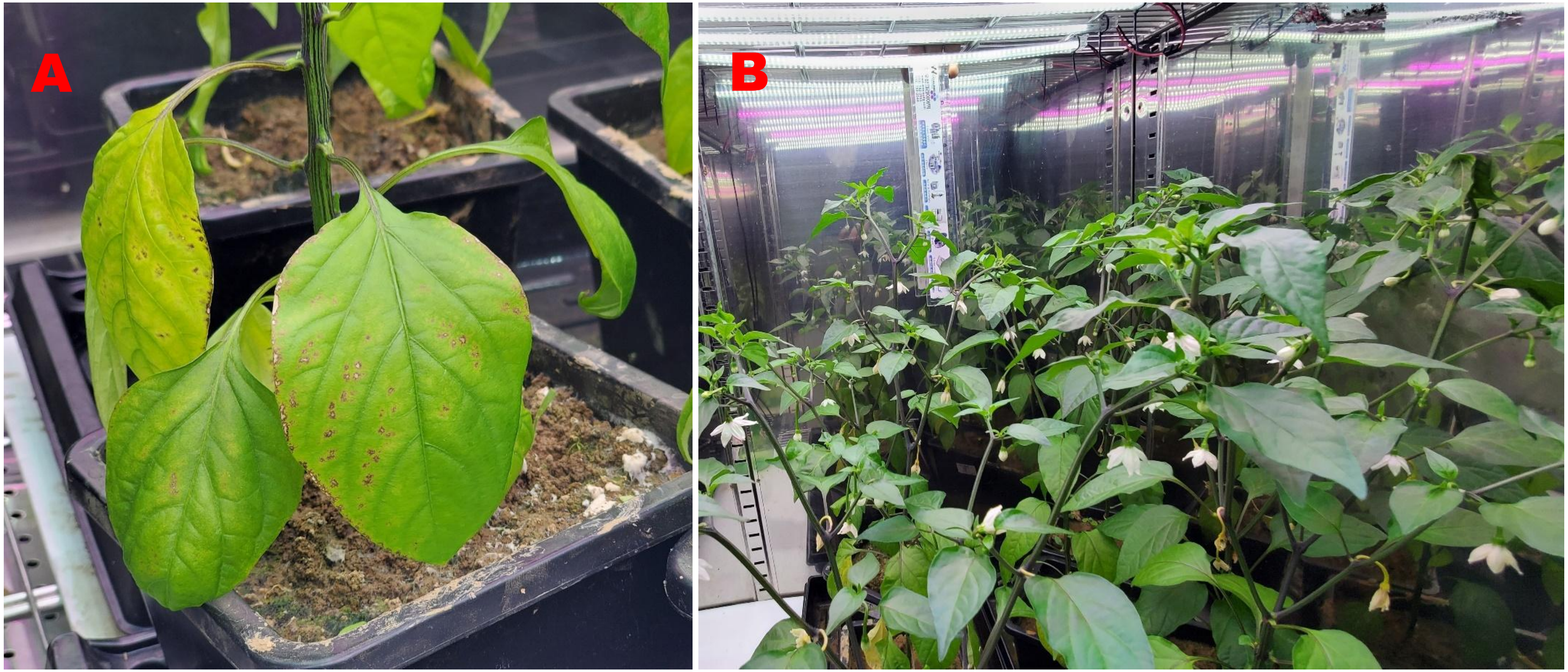

Supplementary Figure S2. Visual comparison of pepper plants treated with SynCom and non-treated controls under *Phytophthora capsici* infection. (A) Non-treated control plants exhibit typical symptoms of *P. capsici* infection, including chlorosis, necrotic lesions, and early-stage wilting. (B) SynCom-treated plants show healthy green foliage with no visible lesions or wilting, indicating improved physiological status and enhanced resistance.
